# The Impact of Group Emotion Regulation Interventions on Emotion Regulation Ability: A Systematic Review

**DOI:** 10.3390/jcm11092519

**Published:** 2022-04-29

**Authors:** Rebekah Moore, David Gillanders, Simon Stuart

**Affiliations:** 1 NHS Greater Glasgow and Clyde, Glasgow Psychological Trauma Service, Festival Business Centre, 150 Brand Street, Glasgow G51 1DH, UK; 2 School of Health in Social Science, Elsie Inglis Quad, Teviot Place, University of Edinburgh, Edinburgh EH8 9AG, UK; david.gillanders@ed.ac.uk; 3 NHS Lanarkshire, Hunter Health Centre, Andrew Street, East Kilbride G74 1AD, UK; simon.stuart@lanarkshire.scot.nhs.uk

**Keywords:** emotion regulation, emotion dysregulation, group intervention, evidence-based practice

## Abstract

Emotional regulation (ER) as a concept is not clearly defined, and there is a lack of clarity about how individuals can improve their ability to regulate emotions. Nevertheless, there is increasing evidence of the importance of ER as a transdiagnostic treatment target across mental health problems. This review examines the impact of ER group interventions on ER ability compared with no intervention, other comparable group interventions, or control conditions. A systematic review was conducted, in which 15 studies were included. Although types of ER intervention were mixed, the interventions had a considerable overlap in skills taught and how ER was measured. In all but one study, the ER intervention improved ER ability. ER interventions were superior to waitlist or treatment as usual, but there was limited evidence to suggest they were superior to other active treatments. Data from some studies suggest that improved ER was sustained at follow-up. Across the studies, there was generally poor linking of theory to practice, which hampers understanding of how interventions were constructed and why different skills were included. Although the results need to be interpreted with caution due to issues with methodological quality with the included papers, there is promising evidence that ER group interventions significantly improve ER ability.

## 1. Introduction

### 1.1. Conceptual Difficulties with Emotion Regulation

Emotion regulation (ER) is an important concept in psychological intervention. There has been considerable growth in publications in this area over the past 30 years, showing conclusive links between healthy ER and good health outcomes and likewise between emotional dysregulation and poor outcomes [1,2,3,4]. ER has been implicated in both the development and the maintenance of a wide range of mental health problems, for example post-traumatic stress disorder, borderline personality disorder, and eating disorders [5,6,7]. ER has also has been proposed as a transdiagnostic factor that may underpin many expressions of psychological distress [1,4,8]. Thus, ER could be an important treatment target in psychological interventions.

One of the key difficulties that remains when designing interventions to improve ER is that of definition. There are myriad definitions and models of ER, and little consensus on what ER actually is [9]. Furthermore, the field is extremely broad, encompassing a range of disciplines including neuroscience, cognitive, social, and developmental psychology. Additionally, it has been observed that there can be a divide between how ER is measured in a clinical context and popular definitions of ER [10]. Models of ER often focus on the process of ER, whereas measurement tools more often focus on trait level-abilities [10]. 

Conceptual clarity about ER is needed to design effective interventions that target ER specifically. Given the lack of conceptual clarity, this review takes a broad definition of ER as meaning the processes and strategies that people use to influence which emotions they have, how they experience them, and how they express them explicitly and behaviourally, in keeping with Gross’s definition of ER [11]. This is a pragmatic choice, allowing the review to include a range of putative ER processes and skills. The review will consider two main models and outline the practical applications of these to intervention and measurement. These models have been chosen as they are widely cited in the field of ER and have good empirical support [9,11,12,13,14,15,16].

### 1.2. A Consideration of the Process Model and the Functional Model of ER

The Process Model is a popular model of ER with considerable empirical support [11,15,16,17,18,19]. It specifies that there are a series of steps involved in emotion generation, and at each of these steps, there are different opportunities for ER strategies. These strategies are used to increase, maintain, or decrease emotional experiences and behavioural responses, depending on the individual’s goal of ER in the specific moment. These steps split ER strategies into five “families” of strategies depending on what point in the process they occur. The five steps are as follows: (a) situation selection, (b) situation modification, (c) attentional deployment, (d) cognitive change, and (e) response modulation [20]. These five families of strategies are then broadly split into antecedent focused (i.e., things people do before the emotion is generated) and response focused (i.e., strategies for when the emotion is already underway). The practical application of this model to date has largely been on identifying what individual strategies are, if they are adaptive or maladaptive, and identifying their relationship to psychological distress and functioning [1,21,22,23]. Much of the research on this model to date has been focused on two strategies: reappraisal (antecedent focused) and suppression (response focused). Reappraisal is a cognitive strategy that aims to modify the meaning and impact of an emotion-eliciting situation, and suppression refers to the act of inhibition of emotional expression (Gross, 1998). Reappraisal is generally seen as an adaptive strategy and suppression a maladaptive strategy: Suppression has been linked to a more negative mood, interpersonal difficulties, and psychological distress, and the opposite pattern has been found for reappraisal [22,24,25,26]. 

A strength of the model and the subsequent work on identifying strategies and their role in psychological difficulties is the ability to target these specific strategies in interventions. For example, as reappraisal is associated with more positive consequences, interventions can teach reappraisal strategies and increase individuals’ use of them. Interventions can also reduce the use of suppression. A specific self-report measure related to these two strategies has been created: the Emotion Regulation Questionnaire (ERQ) [25], which measures the tendencies individuals have to use reappraisal and suppression. The ERQ is widely used and is an example of a practical application of the Process Model as it can be used to measure ER tendencies across a wide variety of applied contexts [22,25,27]. However, it only measures two ER strategies, despite one review [1] identifying six main strategies based on this model: acceptance, avoidance, problem solving, reappraisal, rumination, and suppression. 

ER strategies other than reappraisal and suppression have been under researched, despite, for example, the clearly identified role of rumination within mental health problems [28]. Additionally, the wider process model has been criticised as it is not clear what strategies should be included in ER, and it has also been proposed that the primary function of the strategies may not always be to regulate emotion, so the extent to which these should be considered “ER” strategies is debatable [5]. For example, suppression is widely considered an ER strategy, but inhibiting expression of emotion could be due to the person fearing negative evaluation by others (e.g., crying in public), rather than the strategy being chosen for ER purposes. 

The process model and the ER strategies related to this have also been criticised due to their overreliance on non-clinical samples (often undergraduate students) to verify the theory, as well as using experimental designs that lack ecological validity [4,9]. Furthermore, some studies have shown that relying exclusively on improving adaptive strategies such as reappraisal may be less beneficial than more flexible treatment approaches that target the function of thoughts and behaviours [29]. The model has also been critiqued as the five stages of the model indicate strategies being deployed over time in a set sequence; however, other studies have found that people often utilise multiple strategies simultaneously [30]. 

An alternative to the process model specifically designed for clinical utility is Gratz and Roemer’s Functional Model [14]. This model focuses on the function of emotions, as opposed to the process model’s focus on emotional control and strategies. The Functional Model argues that focusing solely on control strategies may not differentiate between adaptive and maladaptive ER, as what is an effective ER strategy may depend on the context [9,14]. ER in this model is conceptualised as a multidimensional construct involving the following parts: (a) awareness, understanding, and acceptance of emotions; (b) ability to engage in goal-directed behaviours and inhibit impulsive behaviours when experiencing difficult emotions; (c) using strategies flexibly to change intensity and/or duration of emotional responses, rather than to eliminate emotions entirely; and (d) willingness to experience difficult emotions [9]. The model is explicitly designed to be applicable to clinical practice and was developed in parallel with a specific psychometric measure, the Difficulties in Emotion Dysregulation Scale (DERS) [14]. The DERS was designed to assess emotional dysregulation and has an overall score as well as six subscales measuring different aspects of dysregulation: nonacceptance of emotional responses, difficulties engaging in goal-directed behaviour, impulse control difficulties, lack of emotional awareness, limited access to emotion regulation strategies, and lack of emotional clarity [14]. The DERS is widely used with a range of clinical populations. However, its psychometric properties have not been investigated for all of the populations with which it has been used, and as with the ERQ, the initial psychometrics were based on samples of undergraduate students [10,14]. Several studies have found the six-factor structure of the DERS an inadequate fit for different populations, including those with chronic pain, severe mental illness, and those receiving dialectical behaviour therapy (DBT) [12,31,32].

Nevertheless, a range of research shows association between the DERS and symptoms of various psychological diagnoses, indicating evidence of construct validity for the total scale score and the model on which it is based, in which ER is viewed as a transdiagnostic construct [33,34]. An examination of the DERS in a large sample, (*n* = 427) of people seeking treatment for multiple different difficulties and diagnoses found that the total score and five of the subscales (excluding the awareness subscale) provided the best fit for the data and that the DERS may have utility in predicting treatment outcome [10]. Clear strengths of the functional model are that it is designed for clinical contexts, responds to criticism of earlier models such as the process model, and has a specific measurement scale associated with it. Additionally, the functional model’s creators have also developed a group ER intervention based on the model and measurement, an example of clear theory to practice links [35]. However, it is a broad model and therefore remains open to the criticisms outlined above about how ER itself, and ER strategies, are defined [5]. 

Practically, many studies use integrative or blended approaches which acknowledge the contributions and utility of various approaches [36,37]. As noted by Gratz et al. [9], the definition that is used for ER in particular studies seems to be a pragmatic choice related to what type of research question is being asked.

### 1.3. Measurement of ER

As definitions of ER have proliferated, there has been a parallel increase in the development of outcome measures purported to measure ER, the majority of which are self-report measures [1]. Two widely used measures, the DERS and the ERQ, have already been outlined above in relation to their development from specific ER models. 

Measurements of ER are not always related to overarching theories of ER. Additionally, ER scales vary in the construct they aim to measure, for example, a focus on the type of ER strategy used or the frequency of ER strategy use [38,39]. This lack of overarching coherence means it is hard to ascertain whether measures are considering the same underlying construct and, therefore, to what extent different studies using different measurements can meaningfully be compared, contrasted, or synthesised. The lack of clarity around measurement provides a further barrier to understanding which interventions can improve ER. In relation to these issues, there have been small to medium correlations found between aspects of the DERS and the ERQ [33,40,41]. However, other explorations of ER factors have found that process-oriented ER measures (e.g., ERQ) and competency-based measures (e.g., DERS) do not necessarily converge on the same underlying factor, suggesting there needs to be further research and more precise definitions and measurement clarity before different ER scales are assumed to be measuring the same concept [42].

### 1.4. Interventions to Improve ER

The majority of research evaluating intervention efficacy to improve ER has been focused on non-ER specific interventions, such as Acceptance and Commitment Therapy (ACT), Dialectical Behaviour Therapy (DBT), and Cognitive Behavioural Therapy approaches (CBT) [9,43,44,45,46,47,48,49]. These therapies include aspects of ER as part of a wider treatment package but also include other components, so it cannot be ascertained exactly which part of the intervention has an impact on ER or on general clinical or functional improvement. Nevertheless, these interventions do seem to be effective in improving ER when it is measured in various ways (use of strategies, e.g., by way of the ERQ; improving abilities, e.g., measured by the DERS) and improvement in ER as measured in these ways has been shown to mediate improvement in psychiatric symptoms and severity [9,43,46,47,49]. There are other ER-specific interventions that have been developed and found to improve ER, for example, the Unified Protocol (UP) [8], a transdiagnostic intervention, and Emotion Regulation Group Therapy (ERGT) [35], a group intervention for self-harming women with diagnoses of borderline personality disorder (BPD), but research into these is limited [9,50,51]. Where such interventions have been developed for specific populations, the findings may not be generalisable.

There are limited systematic reviews to date that have considered ER as a treatment target. Sloan et al. [4] considered ER as a multifunctional intervention component and included studies using clinical samples that measured ER pre- and post-intervention. The results showed that across intervention type and sample population, both the use of maladaptive emotion-regulation strategies and overall emotion dysregulation were decreased following the intervention in the majority of studies. The review also showed decreases in symptoms of anxiety, depression, substance use, eating pathology, and BPD. Again, as these were multi-component interventions, it is unknown exactly which parts of the intervention improved ER. A systematic review on a transdiagnostic ER intervention showed promising results across a range of disorders, but this only included a single intervention, the Unified Protocol [52], and it was unclear if this ER-specific intervention was superior to interventions that do not specifically target ER [53].

Despite evidence that ER can be improved in a variety of ways, there is still a lack of clarity as to what specific interventions are most likely to improve ER, and research on the utility of ER-specific interventions is still limited. A systematic review of the range of ER-specific interventions in clinical populations will therefore extend knowledge in this area and ascertain which aspects of interventions might contribute to improved ER. Additionally, to date, no review has examined theory-to-intervention links, and this is an important area to consider due to the ambiguity of what ER is and what “form, focus and amount” of ER intervention is needed to improve ER ability [9] (p. 87).

The format of interventions is a further factor that has not yet been reviewed: this is important to consider, given that ER interventions that have been studied are a mixture of individual and group interventions [1,4,53]. Groups can be a pragmatic and resource-effective way of delivering interventions, with the added benefit of normalisation, reducing shame and providing opportunities for validation [54]. Given that ER appears to be an important target for treatment across a range of psychological difficulties, group formats are well suited to ER interventions; furthermore, because therapeutic-group interventions are often clearly boundaried in terms of session numbers, topics, and materials, it may be clearer for reviewers to identify exactly what an intervention consists of. For this reason, the decision was made to focus exclusively on groups in the present review. 

### 1.5. This Systematic Review

To address the gaps outlined, this systematic review will focus on specific ER groups in adult clinical populations and the impact of those groups, as determined using measures of ER. It is well established that ER interventions can improve specific psychopathological symptoms and general distress and wellbeing. This review will therefore focus on measures of ER as a primary outcome. A secondary focus will be on how ER is defined and how this leads to the intervention choice. It is hoped that the review will be a useful addition to the evidence base for treatments to improve ER, and help to clarify how these can be theoretically driven. The review will also examine and describe the quality of evidence of research in the field. 

Review questions:

1. What is the impact of ER groups on ER skills in adult clinical populations?
2. What ER theories, definitions, and concepts are used in these interventions?
3. How strong are the theory-to-practice links in these intervention groups?

These questions are broad, reflecting the current state of the literature as outlined in the Introduction. While the breadth and diversity of the papers included mean there are some elements of a scoping review, our approach to searching, inclusion and exclusion, data extraction, and quality assessment is entirely systematic, as detailed in the Methods section below.

## 2. Materials and Methods

The systematic review was conducted in accordance with PRISMA guidance, and a protocol for the systematic review was registered on PROSPERO, ID: CRD42020196080. 

### 2.1. Inclusion and Exclusion Criteria

Studies were included if they were a group-based intervention specifically designed to improve emotional regulation; if they had pre- and post-outcome measures on a validated scale of emotional regulation (e.g., DERS); and if the participants were adults from a clinical population. For the purposes of this review, a clinical population is defined as people who have a mental health diagnosis or who score above clinical cut-offs on validated measurements of psychological distress or dysfunction, and are seeking or receiving treatment for their mental health difficulties. Due to time and funding constraints, only English-language publications were included. All types of intervention study designs were included, for example, randomised control trials, non-randomised control trials, single-case experimental designs where sufficient data were available, case series where sufficient data were available, pilot studies, service evaluation studies, and pre–post treatment efficacy studies. 

Studies were excluded if the participants were under 16 or over 66, if they were from a non-clinical community population; or if the intervention was part of parent-, couple-, or family-based interventions. Interventions that were not clearly specifically focused on ER or did not have a majority of sessions focused on ER (defined as over 50% of sessions focused on ER) were also excluded. Interventions that were focused exclusively on people with cognitive impairments, intellectual disabilities, autism spectrum disorder, or attention deficit hyperactivity disorder were also excluded. Studies with only qualitative data were excluded, as were trial registers and all non-peer-reviewed research including doctoral theses, MSc projects, conference proceedings, books, reviews, and meta-analytic studies.

### 2.2. Search Strategy

Three databases were used: PsycINFO, MEDLINE, and Embase. Reference lists from included studies were hand searched, and a hand search of Google Scholar was also performed to identify additional literature not captured through the database searches. The search terms that were used were: emotion* regulation or affect regulation or emotion* dysregulation or emotion* efficacy or emotional predictability or STEPPS or DBT or Unified Protocol or Dialectical Behavior Therapy or Dialectical Behaviour Therapy AND Skills or course or training or intervention or treatment or therapy AND group. The search was performed on 17 July 2020 and repeated on 1 April 2021 and 28 October 2021 to check for any additional papers for inclusion. 

### 2.3. Data Extraction

**Figure 1:**
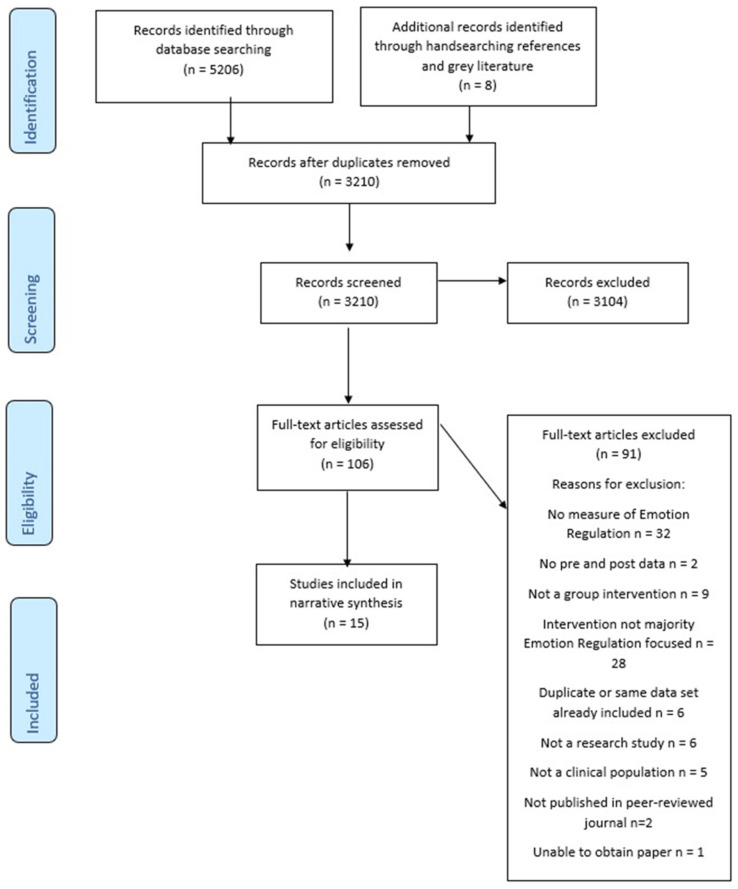
PRISMA 2009 Flow Diagram [55].

The first author screened titles and abstracts against the inclusion and exclusion criteria. Full-text versions of studies that were deemed to meet the inclusion criteria were then examined and criteria applied. Figure 1 shows how the final number of included papers were selected and the reasons for exclusions. Relevant data from each study were extracted by the first author and are presented in tables in the results section.

### 2.4. Quality Assessment

Once the data were extracted, this study used a modified version of the Downs and Black checklist [56] (see Appendix A). The modifications were in response to criticism of the checklist and in relation to the specific study aims [57,58]. This included adding questions about whether studies stated an ER theory, assessing group equivalence at baseline, and controlling for concomitant treatment. The Downs and Black is a widely used checklist and has strength in being able to accommodate different study designs [56,57,59]. Score ranges are given corresponding quality levels: excellent (34–38); good (26–33); fair (19–25); and poor (≤19) based on Hooper et al.’s (2008) [60] description of quality level ratings. 

The first author rated all papers for quality using this checklist, and final author rated 30% of the papers that were selected through random number generation. From this, inter-rater agreement was calculated. 

### 2.5. Data Synthesis

All papers had sufficient data that could be extracted. A narrative synthesis was decided to be the most appropriate way of summarising the included studies, due to the heterogeneity in intervention type, sample population, and study designs. For these reasons, a meta-analysis was not deemed appropriate. 

## 3. Results

### 3.1. Characteristics of Included Studies

A total of 15 studies were included in the narrative synthesis. Table 1 illustrates the study characteristics.

The study designs were varied. Six were randomised control trials [35,61,62,63,64,65]. Of these, one had an active comparison intervention as well as a non-active waitlist control condition [61], one had an active control only [64], and four had non-active waitlist or treatment as usual (TAU) controls [35,62,64,65]. In this context, an “active control” condition means the participants received a non-ER based psychological group intervention, and “non-active waitlist control” means participants in this condition were on a waitlist to receive the ER intervention but during the study period did not receive any psychological group intervention. Two further studies had randomisation processes and control groups but were described as either pilot studies or quasi-experimental studies [66,67]. Of these, one had TAU controls [67] and one had active comparators as controls [66]. Five studies were “before and after” (B&A) studies with no control or comparison group [34,36,68,69,70]. One study was a B&A study with an active comparison group [71], and one was an observational case series study [72].

In the studies with active control conditions, the type of intervention used was varied. Three used a generic supportive and/or psychoeducational group with no ER skills to control for the common therapeutic factors often found in group interventions [61,63,66]. Two used different combinations of DBT skills modules including mindfulness, interpersonal effectiveness, and ER [66,71].

There was considerable heterogeneity across the types of ER intervention used. Apart from one study with twice weekly sessions [69] the sessions were all delivered once a week. The number of ER sessions ranged from 5 to 14, with the mean number of sessions being 10. Session length ranged from 75 to 180 min, and the mean session length was 107 min.

There was a range of target populations in the studies. Four studies recruited people with a diagnosis of Borderline Personality Disorder (BPD) who self-harmed [34,35,64,70], and one study also recruited people with a diagnosis of BPD but did not stipulate self-harming as one of the criteria [66]. Three papers were transdiagnostic [36,62,71]. One paper focused on participants with substance use problems [65], and two focused on those with post-traumatic stress disorder [63,72]. One paper only recruited people with major depressive disorder [61], one paper recruited people with social anxiety [67], one paper focused on people with eating disorders [68], and one paper recruited people with psychosis [69].

The gender of participants was disproportionately female. Eight studies recruited only women [34,35,63,64,66,67,68,70], and one study recruited men only [65]. In four out of six studies with mixed gender, women represented the majority of participants, ranging from 58% to 81% of the sample size [36,61,62,71]. In the mixed gender studies, two studies had a majority of male participants: one where women represented 17% of the sample [72], probably due to the sample population being veterans with PTSD. The other [69] and was closer to an even distribution with 40% women. Additionally, for the mixed gender studies, it is unknown the proportion of men and women to whom the intervention was offered, so it is similarly unknown to what extent the proportion of men/women in the sample represents the wider population or if it is a self-selecting sample where more women have chosen to opt in to the intervention.

Age in the studies ranged from 18 to 69 years. Two studies focused on young people, with age ranging from 18 to 29 [68,71]. Mean ages in the studies that reported this range from 24 to 47.

### 3.2. Quality Assessment

Six studies were rated as poor using the modified Downs and Black [56] checklist [34,36,67,69,71,72], six were rated as fair [35,64,65,66,68,70], and three were rated as good [61,62,63]. No studies were rated as excellent. Papers rated good or fair are higher quality, and results from these papers are at less risk of methodological biases. Papers rated poor have a number of methodological problems meaning findings from these papers are less trustworthy. Particular issues that the quality checklist highlighted will be explored in the next section.

Inter-rater reliability was calculated using Cohen’s Kappa, κ = 0.524, *p* < 0.005, which indicates there was a moderate level of agreement between the two raters. Differences in scoring were resolved through discussion.

### 3.3. Missing Data

All studies had at least one point of missing data and varied in approaches to managing this. In intervention research, intent to treat (ITT) analysis and imputation methods are commonly used to manage missing data while preserving sample size and power. ITT measures the effect of the intervention on all participants who were assigned to the study, regardless of whether they subsequently dropped out or did not complete the intervention [73]. ITT is thought to give a pragmatic estimate of the benefit of treatment interventions, and not using this may cause the clinical effectiveness of an intervention to be over-estimated [74]. Where data are missing, this is normally managed in ITT by using imputation methods, usually by carrying forward the last observed response [75,76]. ITT is thought to give a less biased estimate of treatment effect than completer analysis, and this is reflected in the modified quality checklist, which has questions about how missing data were handled.

In the studies in this review, eight used ITT analysis [34,36,61,62,64,66,70,71]. Apart from one paper [36], which excluded five data sets from participants who had been found to meet the exclusion criteria during the study, papers using ITT analysed all participants that were assigned to the intervention and control groups.

For the papers that did not use ITT, only one paper described a method of handling missing data [69]; in this study, the outcome data were analysed and determined to be missing at random. The other papers analysed only data from participants who completed the study and provided outcome measures at the last time point specified. Where missing data are minimal, using completer analysis may not introduce bias, which was the case for two studies [35,67]. As missing data increases, so does the likelihood of bias. One paper had over 50% of the data missing, meaning interpretation of these findings needs to be conducted with caution due to the high risk of bias and overestimation of treatment effect [72]. The remaining three papers using completer analysis had around 10% of the data missing, which may affect the analysis and introduce bias into the results [63,65,68].

### 3.4. Power

Related to the issue of managing missing data, statistical power is another area that many papers did not address. If studies are not adequately powered, the probability of a type II error is increased, as well as type I error as the estimate of effect is not as reliable as if it were made with more participants. Power analyses are ideally performed a priori and help the study determine sample size needed to detect statistically significant effects. Five papers considered power when setting the sample size and were adequately powered at analysis [61,62,63,65,70]. One paper considered power issues post hoc and concluded that their study was under-powered to detect even large effect sizes [66].

For papers with no or limited information about power, the first author performed calculations using G*Power [77] to ascertain whether studies were likely to have had adequate power to detect medium sized effects or larger. For pre–post studies with no control condition, two papers are likely to be underpowered to detect a medium effect size (within participants), increasing the chance of type I and type II error [34,68]. The other three were sufficiently powered [36,69,72]. For studies with control groups, three studies were likely underpowered to detect between-group effects and within x between group interactions with medium-sized effects [35,67,71]. One study was underpowered to detect between group differences but adequately powered for detecting within x between interactions [64].

Overall, eight papers were thought to be sufficiently powered, resulting in considerable risk of type II errors, and the potential for type I errors in seven of the included studies.

### 3.5. Concordance with Intervention

Whether the intervention was reliably delivered as intended is a further quality marker. Factors that may affect this include participant concordance with intervention, for example, if they attended all sessions, as well as facilitator fidelity to the treatment intervention. Knowledge of facilitator training and facilitator competence may also give information on whether the intervention was delivered as intended.

Four studies contained no information about therapist competence or training, fidelity to intervention, or participants’ concordance with intervention, meaning it is unknown the extent to which the intervention was delivered as planned [35,65,67,72]. All other studies provided information that indicated group facilitators were competent and sufficiently trained and supervised on the interventions they delivered. Seven studies examined intervention fidelity, and apart from one paper [62], which did not describe the outcome of fidelity checking, facilitator adherence to the intervention manual was high, indicating the intervention was delivered as intended [34,61,63,64,66,70].

Only three studies recorded the number of sessions attended by participants [63,69,70], and in Sahlin et al. [70], the number of sessions attended was associated with greater treatment effects, indicating the importance of participant concordance with intervention. However, in Ford et al. [63], treatment concordance was shown to be unrelated to improvements in ER. Ryan et al. [69] did not examine if session numbers had any relationship to outcome. Two papers asked participants to leave the intervention if they missed more than one session, implying that included participants attended all but one session [36,62].

### 3.6. Impact of Other Interventions 

Controlling adequately for the impact of other interventions out with the study protocol is an important quality feature. If studies have not adequately described and accounted for other non-study interventions, it is impossible to ascertain the impact of the ER intervention on ER outcome measures, as it may be confounded by the impact of non-study interventions. Two papers explicitly excluded any participants who were currently receiving any other therapy, controlling for this possible confound [61,62]. Five papers had no information on whether any other treatment was being received by participants [36,63,67,71,72]. When looking at the settings of these studies, it is possible that there may have been other ongoing treatment, especially as referrals to the ER interventions often came from other providers (nurses, psychiatrists etc.) who may have been continuing treatments with participants. In the remaining seven studies, participants were described as receiving a range of other treatments. Receiving other treatments is less likely to impact the results if studies have a control group also receiving equivalent amounts and types of non-study interventions. In four papers, participants were receiving other interventions, but there was little to no information as to what these interventions were and if they were equivalent across study groups, so it is difficult to assess their impact [65,66,69,70]. Sahlin et al. [70] and Ryan et al. [69] also had no control group, meaning there is potential for the outcome data to have been impacted by other interventions to an unknown extent. Two studies gave a specific outline of what other treatment was received by participants, and as the control group in these studies was treatment as usual and was shown to be equivalent across intervention and control groups, this possible confound is well controlled for [35,64]. Finally, in two studies, the other interventions which participants were receiving were better described, but as they had no control group, it is unknown to what extent non-study interventions may have impacted outcome data [34,68].

### 3.7. ER Intervention and ER Theory

ER treatment interventions drew from various different treatment modalities, mainly from ACT, DBT, and CBT, as well as UP, mindfulness interventions, and emotion-focused psychotherapy. Most studies used established group ER treatments, for example, the ERGT, but a minority were studies evaluating newly developed interventions. 

The most commonly delivered intervention was Emotion Regulation Group Intervention (ERGT), used in four studies [34,35,64,70]. ERGT is an acceptance-based behavioural group designed to reduce self-harming behaviour in women with a diagnosis of BPD [35]. Two studies used the ER module from DBT skills training [66,71]. One study [61] used affect regulation training, a transdiagnostic intervention designed to enhance emotion regulation skills in clinical and at-risk populations. One study used emotion regulation group therapy based on the Gross model of ER (ERGT(G)) [65]. Two studies used a group version of the Unified Protocol, and one used Emotional Schema Therapy, both transdiagnostic interventions [62,67,72]. Four studies were evaluating a newly created group intervention, and as with the established group interventions, drew on a variety of established therapeutic modalities and existing ER groups to create the new intervention [36,63,68,69]. 

Despite the variety of approaches, the content of each intervention had considerable overlap. Core components across groups were psychoeducation about emotions, strategies to increase emotional awareness, identifying and labelling emotions, accepting and tolerating emotions, promoting appropriate expression of emotion, monitoring emotions and reactions, and understanding triggers for emotion. Mindfulness was another core component across many groups, specifically with the focus of creating distance from emotions and cultivating a non-judgemental attitude to them. 

Most groups were proactive skills groups and taught a variety of specific skills to enable participants to modify their emotions and emotional reactions. There was some variation across groups in the specific skills, but the broad categories of skills frequently implemented were reappraisal, relaxation, grounding, distraction, problem-solving, self-soothing, acting opposite, and increasing opportunities for positive emotions (e.g., by way of self-care). Many groups emphasised the importance of skills practice outside the group and gave participants homework and practice tasks between sessions. A minority of groups included active emotional exposure in the group sessions [36,62,72]. Some groups had other content not specifically focused on ER, for example, psychoeducation on specific psychological disorders [35,63,69] or a focus on values [68]. This non-ER content made up a minority of sessions. Interventions with a strict focus on only ER skills were Bacon et al. [36], Dixon-Gordon et al. [66], Morvaridi et al. [67], and Rizvi and Steffel [71].

As outlined in the introduction to the present review, there are numerous theories and models of ER. Although there was considerable commonality in core skills and tasks across groups, there was less commonality or clarity relating to why particular skills were included and which theories were being drawn upon to guide the creation of the intervention. One paper did not define ER at all [70]; one paper described ER only as a treatment target in people who had experienced trauma and/or incarceration [63]; two stated that emotional dysregulation was the root cause of deliberate self-harm [34,64]; and four stated that skills deficits in ER underlie and lead to a range of mental health problems [61,62,66,71]. These eight papers did not define what ER was or how it might lead to the intervention chosen in the studies.

A clear definition of ER was outlined in seven studies. These papers drew on a range of models and theories. One paper [65] focused solely on the Gross process model [20], which gives a range of different targets for ER unfolding over time. One paper [67] drew on a social-cognitive, meta-cognitive approach to emotion regulation as described by Leahy [78], which states that individuals have differing abilities in identifying, labelling and differentiating their emotions, their appraisal of their emotions, and their tendency towards unhelpful coping strategies. Two papers [35,72] used Gratz & Roemer’s [14] multidimensional definition which emphasises the functionality of emotion regulation. The remaining three papers created an integrative model and theory of ER from a range of sources. Bacon et al. [36] drew on the Gross process model [20] but also included Koole’s functional approach [3], emphasising the importance of functions of ER, as well as the theory of constructed emotion [79]. Holmqvist Larsson et al. [68] combined Gross [20] and Gratz and Roemer’s [14] models and also emphasised the importance of ER in the development and maintenance of eating disorders. Finally, Ryan et al. [69] conceptualised ER as a set of regulation strategies that can be adaptive or maladaptive and the importance of these maladaptive strategies as a mechanism that maintains psychosis.

Regardless of ER definition, many papers acknowledged the importance of ER as a transdiagnostic concept underlying and driving emotional distress across mental health problems [36,61,62,68,69,71,72]. Five papers had clear theory to practice links, where the theories and models of ER clearly informed the intervention that was used in the study [35,36,65,67,72].

In relation to the quality of studies, it seems that clear definitions of ER do not match to quality labels. A study may be methodologically “good” or “fair” quality but may not describe ER well or have good theory to practice links [61,66]. Conversely, some papers rated as “poor” quality provided very thorough description of ER theories and how these lead to intervention targets [36].

### 3.8. ER Outcome Measures

The majority of included studies (12 out of the 15) used the Difficulties with Emotion Regulation Scale (DERS) as a measure of emotional regulation [14]. This scale is widely used, well validated, and has significant positive association between scores on the DERS and symptoms of various mental health problems, providing support for the idea of ER as a transdiagnostic concept [10,33,80,81]. 

Other ER measures used in the studies were the Emotion Regulation Skills Questionnaire (ERSQ) [61], the Generalized Expectancies for Negative Mood Regulation (NMR) [63], and the Emotion Regulation Questionnaire (ERQ) [62,67]. Some studies have shown that the ERSQ correlates well with the DERS and NMR [82], and the ERQ and DERS have small to medium correlations between aspects of their scales [33,41]. This provides some evidence that these four measures assess similar constructs of emotion regulation and can be compared. However, other studies have found that ER measures based on different theories, such as the DERS and ERQ, do not always converge on the same underlying factor, suggesting there needs to be caution when comparing different outcome measures [42].

### 3.9. Outcomes

Table 2 summarises the main outcome data from the included studies. With the exception of one paper [63], the ER intervention significantly improved ER ability from pre to post treatment. Additionally, in all papers, change in ER ability was also accompanied by changes in other clinically relevant measures, including reduced self-harm frequency and diagnosis-specific symptomatology. 

Considering within participant effects, for studies showing a pre–post improvement in the ER outcome measure, there was a large effect size in nine cases [6,34,35,36,62,67,68,70,71]; a medium effect size in three [61,64,72]; and a small effect size in two [66,69].

This must be interpreted in light of the quality ratings, and in papers rated “good”, one found no significant effect of the ER intervention, one had a medium effect size, and one had a large effect size [61,62,63]. For papers rated “fair”, three found a large effect size [35,65,68], one a medium effect size [64], and one a small effect size [66]. It seems that papers rated as “poor” were more likely to find large effect sizes, with five out of the seven papers reporting a large effect size [34,36,67,70,71]. This is considered further in the Discussion below.

The four studies using a range of comparison interventions, including different DBT skills modules and common factor controls, found no difference between the ER intervention and the comparison intervention [61,63,66,71]. Although one study [71] did not find a significant difference, the active comparison in this case was made up of two of the four skills sections that commonly comprise a DBT intervention (the four skills sections are Mindfulness, Distress Tolerance, Interpersonal Effectiveness and Emotion Regulation). The two skill sections in the active comparison in this case was ER and mindfulness, and the ER intervention was the ER skills section from DBT alone. This indicates that the ER skill section may be a more “active” part of the intervention as the addition of mindfulness made no significant difference to outcomes. In the five studies comparing ER interventions to waitlist or TAU, there were significant differences between the ER and control condition, indicating the ER was superior to waitlist and TAU [35,62,64,65,67]. It would seem from these results that there is reasonable evidence that ER interventions can improve ER skills with small-to-medium effect sizes in higher quality studies and are superior to waitlist or treatment as normal, but the evidence that they are superior to other interventions is limited and inconclusive.

The majority of papers only collected data pre- and post-intervention. Of the six that collected follow-up data, the follow-up period ranged from two weeks post treatment to nine months [61,64,66,69,70,71]. The lack of follow-up and variability in follow-up times makes it hard to assess the long-term impact of ER interventions. Nonetheless, from the data available, ER improvements were either maintained at follow-up [61,64,70,71] or continued to improve [66,69]. These results need to be interpreted with caution due to the heterogeneity of follow-up times and four of the papers including follow-ups being rated as poor quality.

## 4. Discussion

This review focused on three key questions:

1. What is the impact of ER groups on ER skills in adult clinical populations?
2. What ER theories, definitions and concepts are used in these interventions?
3. How strong are the theory-to-practice links in these intervention groups?

In relation to the first question, from the results, it seems that there is good evidence that ER ability can be improved, at least in the short term, by ER group interventions across a range of ER intervention types and populations. This is consistent with other evidence in the field, i.e., that changes in ER seem to occur no matter what the ER intervention is, the ER construct examined, or the sample population [4,53]. Effect sizes, where it was possible to calculate them, ranged from small to large, with the majority being large. ER interventions were found to be superior to waitlist or TAU; however, it is not clear from this review if ER is any better than any other group intervention not specifically targeting ER, or if skills can be improved in the long term by these groups. This conclusion is similar to Sakiris and Berle’s review [53], where it was also unclear how UP, an ER-specific intervention, performed compared to other interventions. The present review gives provisional evidence that gains made in treatment can be maintained, but due to heterogeneity in follow-up times and poor quality of some studies, this is not certain. Future studies should, where possible, include follow-up periods of at least 6 to 12 months to ascertain the longer-term impact of ER group interventions. 

The effect sizes also need to be interpreted in the context of methodological quality, and this review found studies that were assessed as being poor in methodological quality were more likely to find larger effect sizes compared to those rated fair or medium. This finding of effect sizes increasing as study quality lowers is a common finding in psychological therapy research [83,84]. Methodological issues such as underpowered studies, lack of an adequate control, or other confounding variables are more likely to be present in lower-quality studies and may lead to an overestimation of intervention effect size. 

In relation to the second and third question, half the studies did not define ER or state a theory of ER upon which the intervention was constructed. Given the myriad definitions of ER in the field, and wide disagreements on what ER is and is not, this may be problematic as it is unknown how and why skills are being taught. Depending on what theory is being drawn on by facilitators, the same skill may be delivered in very different ways or for different purposes. For example, relaxation exercises are widely included across ER interventions, but the purpose and function of these exercises may differ according to the model of ER. Poor theory to practice links further complicates the ER field and undermines efforts to produce truly evidence-based interventions where interventions are designed based on theoretical models. These developments are needed to establish more targeted and efficient interventions that best meet individual and service needs. Future research in this area should focus on clearly defining ER, and should demonstrate theory to intervention links and accurate measurement of ER. Unfortunately, this is not a new proposal. More than a decade on from a similar call for conceptual clarity [85], the field seems to be no further forward in this regard. This review reiterates these findings and emphasises the importance of having links between affective science research and clinical practice. Nevertheless, although only present in a minority of studies, it is encouraging to see some studies that do have clear theories that inform the ER intervention [35,36,65,67,72], and this enhances our understanding of what improves ER. It is also interesting to note that many papers are now using integrative models of ER, perhaps reflecting the wider state of the field of ER, where ER is seen as complex and multi-faceted, and moving to a “both/and” position rather than trying to prove one theory over another. However, it is worth considering that such integrative models may be a double-edged sword. Although they may bring benefits in terms of combining strengths of different models, and making definitions of ER more inclusive, the integration of possibly contradictory models may further dilute the clarity of ER conceptualisation, clear measurement, and clear theory to intervention links. 

To move the understanding of ER and how it can be improved, it is also necessary to have clarity in how ER should be measured. As stated earlier, various ER measurements exist, but it is unclear to what extent they actually measure the same underlying construct [42]. The majority of included studies used the DERS, and this seems to reflect the wider field of ER, where the DERS is frequently used as the default ER measure. The DERS is widely used and is largely well accepted [10,86]. However, what it measures is emotional dysregulation, rather than emotional regulation, and it does not measure any aspect of ER strategy use, as the ERQ does. There has also been criticism of the DERS based on the awareness subscale not contributing to the higher-order total score of the measurement and problems with accurate scoring due to some items requiring reverse scoring [12,40,87]. A modified version of the DERS, where the reverse scored items are reworded, seems to effectively manage both of these difficulties [87]. This version should be used in future to prevent methodological difficulties in scoring and to retain psychometric strength. 

Related to ER strategy use being an important construct in ER, a recent exploratory factor analysis of various ER measures revealed three different overall factors: out-of-control negative emotion, emotion awareness and expression, and cognitive strategies [42]. While the DERS measures the first two factors, it does not measure the use of strategies. Therefore, it may be helpful in future research to use multiple ER measures that cover these three factors, for example, the DERS plus a measurement of strategy use. 

This is the first review of group interventions of ER and has shown that, regardless of intervention and measurement type, ER was improved. This suggests that ER can be improved in multiple ways, an important finding clinically as services may be limited as to what ER intervention they can provide, because of factors including but not limited to staff availability, training and managerial priorities. The sample populations were varied, and again this shows the importance of ER as a transdiagnostic treatment target across psychological difficulties, and can give confidence to clinicians delivering ER interventions to people presenting with a range of psychological difficulties. 

The focus on ER definitions and theory, and their links to the intervention, is a further strength as it has not previously featured in reviews, and it has been observed there may be a disconnect between theoretical models and clinical application [10]. As stated previously, this disconnect means the understanding of what ER is and how it can be improved is not currently well understood academically or clinically. Greater conceptual clarity regarding ER is likely to lead to more precisely targeted interventions that improve patient outcomes. Therefore, adding the consideration of theory-to-practice links in assessments of studies quality, as this paper did, may be helpful in future reviews. 

The review supports the suggestion that, across a range of different ER interventions, there seem to be common components that are mediating the changes in ER. The next steps in research may involve dismantling studies of ER interventions to try to establish exactly which parts of the intervention are mediating changes in ER measures. For example, there is some evidence from this review that mindfulness may be a less “active” component than other ER skills [71], and dismantling studies could explore this further. Relatedly, factor analytic studies could further clarify the measurement components of ER, and longitudinal modelling studies could track changes in components of ER in response to specific interventions. One of the difficulties that such research may face, and brough to light by this review, is that what the intervention actually involves can be hard to ascertain from the article. Some studies were very clear on what was delivered week to week, and some also referred to published manuals or previous articles with more information. Others had very little information on what was included in the intervention. This is a problem common in psychological intervention research [88] and may in some instance be due to word limits on journal submissions, but authors should clearly outline the intervention, or provide supplementary online materials and, if needed, refer to manuals or previous articles. 

An alternative way of addressing the issue of “active” parts of the intervention would be to construct ER interventions based on skills that had been independently verified as being effective at improving ER, as one of the included studies did [36]. This was in response to criticism of previous ER groups that were created by selectively extracting parts of individual treatment approaches: while such an approach as a whole may have evidence of efficacy, it is unknown if the selected parts are efficacious on their own [36]. It may be that using both these top-down and bottom-up approaches to constructing effective ER interventions could be considered for future research. 

A further finding is that ER can be improved in a relatively short period of time in a group setting, with 10 weeks as the mean length of interventions in the studies reviewed. This is important clinically given the pressures of services to meet demand for psychological interventions. However, further research is needed to understand which ER interventions have the most impact, if they are superior to other generic interventions, and if the impact is sustained long term. 

A potential limitation to this review is the largely poor quality of included studies as rated using the Downs and Black modified checklist [56], which limits the strength of the findings. The oldest paper included was published in 2006, suggesting the evidence for group interventions for ER is still fairly young, so it is perhaps unsurprising that many studies are low in quality as the evidence base for ER interventions is still being established at this time. Many of the included studies label themselves as pilot studies and are likely more concerned with establishing initial evidence for a particular intervention than on methodological quality. As the field develops further, the quality of studies needs to be a particular focus to improve the standard of evidence of ER interventions, and methodological shortcomings outlined in the results must be addressed. Particular weaknesses across studies were considerations of power, how missing data was handled, and measurements of concordance with the intervention. A further limitation in relation to quality is that not all papers were independently rated by the second rater. Although second rating a proportion of the papers provides an estimate of the first rater’s accuracy, it is not as robust as having all the papers independently rated. 

The review was inconclusive as to whether ER-specific groups were more effective at improving ER than other generic group interventions, and further research is needed to understand this. Because of the impact of non-specific group effects [4], it is important to have active controls that account for these findings to further develop knowledge of how ER can specifically be improved. Two higher-quality studies [61,63] used generic group interventions in this way. Future research should use similar interventions as comparisons to ascertain the specific effects of the ER intervention. 

One potential limitation of the present review is that the studies included are heterogeneous in terms of intervention type and sample population, making it is harder to compare their outcomes. However, this presents an accurate representation of the state of the research on ER interventions and reflects how ER is being applied in a clinical context: i.e., there is considerable variability in how ER is conceptualised and what interventions involve. In the future, when there is more research on specific ER interventions, it may be possible to have reviews and meta-analyses with more homogeneous samples, interventions, and measurements and make comparisons between different ER interventions. 

Most participants across studies were women, with eight studies recruiting only women. This may limit the generalisability of the findings to men but also may reflect wider issues in the field and in society. Women tend to be over-represented in mental health services and are more likely to be diagnosed with “emotional” disorders such as BPD [89,90]. In contrast, men may be reluctant to seek help due to assumed stigma and are more frequently diagnosed with substance use or aggression related problems [91,92,93]. This reflects wider cultural norms on emotion and gender, where women are expected to show more of their emotions such as sadness, happiness, and emotional instability, and men are expected not to show their emotions, other than those such as anger, which is more socially acceptable for men to express [94]. Interestingly, the clinical literature shows that both men and women are likely to have difficulties with ER, suggesting a need for treatment regardless of gender [95]. Where men were recruited in the studies, they were generally in the minority of participants, and this may reflect a self-selection bias, where interventions labelled in some way as an “emotional regulation” group may be less attractive to men due to societal perspectives on gender and emotion. Future studies should examine to what extent the findings of this review are applicable to men taking part in ER interventions. Additionally, it may also be helpful to examine whether factors related to how a group is labelled and advertised influences who decides to take part. 

A further factor potentially impacting the generalisability of the findings is ethnicity. Where studies described ethnicity, it was overwhelmingly white. This is a common problem in intervention research in Western countries, where very few interventions include racial and ethnic minorities in efficacy studies [96]. Given the well-established health inequalities faced by racial and ethnic minorities in these societies, and the often poor treatment outcomes with mental health care, it is essential that future research conducted in Western countries considers strategies that ensure recruitment of people from minority ethnic backgrounds [97]. However, two of the included studies in the present review were conducted in Iran, and although no ethnicity data were presented in these studies, it can be assumed that the sample were predominantly Iranian nationals [65,67]. This indicates that ER interventions have efficacy outside white Western populations. 

One confounding variable in many of the included studies was the impact of non-study interventions. The majority of studies did not exclude participants who were receiving other psychological intervention or did not specify whether other psychological intervention was being received. This is an obvious confound, as if participants were receiving other psychological interventions during the ER intervention period, it is impossible to know if the change in ER scores is due to the ER intervention or non-study intervention. Although there can be practical and ethical difficulties with achieving full control of concomitant interventions, future studies should attempt to control for this confound, either by ensuring equivalence of other treatment across groups as outlined by Gratz et al. [64] and Gratz and Gunderson [35] or by including it as an exclusion criterion as Berking et al. [61] specified. 

Due to the timescale and resources of this project, only English-language studies were included. This may mean studies in other languages are missing from the review, but it is encouraging that included studies come from a variety of countries spanning three continents. 

## 5. Conclusions

To the authors’ knowledge, this is the first review examining the impact of group ER interventions on ER in clinical populations. It builds on the existing evidence base of how ER can be effectively improved. However, the studies had a considerable amount of heterogeneity and were on the whole poor in methodological quality, limiting the confidence in the findings. Additionally, although there was overlap in skills taught across interventions, on the whole theoretical underpinnings of the interventions were only well described in a minority of studies. To advance the understanding of how ER can be effectively improved, there needs to be a clearer conceptualisation of ER, good theory to practice links, and consistent measurement. These developments are likely to lead to more effective and targeted interventions to improve ER, which will ultimately improve treatment outcomes for people presenting with psychological distress.

## Figures and Tables

**Table 1:** Summary of study characteristics.

Study	Design	Intervention(s)	Intervention Duration	Study Population and Demographics	Sample Size and Gender (% Female)	Data Used for Analysis	Data Collection Points
Bacon et al. (2018) Scotland	Before and after Randomisation: No Control group: No	1. Semi-manualized ER group programme	6 weeks, 150 min each session	Patients attending routine secondary care mental health services, variety of diagnoses Mean age 1. 36 No range reported	*N* = 52 (81%) Number in each condition 1. *N* = 52	Total data sets analysed *N* = 47–30 full data sets and 17 Intent to treat (ITT) data sets	Pre and post intervention No follow-up
Berking et al. (2019) Germany	Randomised control trial (RCT) Randomisation: Yes Control group: Yes, and active treatment comparison	1. Affect regulation training (ART) 2. Waitlist control condition 3. Common factors control condition	6 weeks, 180 min each session, then four weeks of independent skills practise and one 90 min booster session on week 8	Diagnosis of major depressive disorder Mean age 1. 38.9 2. 38.8 3. 41.1 Range 18–69	*N* = 218 (64%) Number in each condition: 1. *N* = 76 (64%) 2. *N* = 72 (67%) 3. *N* = 70 (61%)	Total data sets analysed *N* = 218–181 completed treatment and 37 ITT data sets	Pre, mid, mid, post intervention Two weeks post-booster follow-up
Corpas et al. (2021) Spain	Randomised control trial (RCT) Randomisation: Yes Control group: Yes	1. Brief Group Transdiagnostic Psychotherapy (based on UP protocol) 2. TAU–pharmacotherapy only	8 weeks, 60 min each session	Mild/moderate clinical symptoms of somatoform, anxiety and/or depression disorders Mean age 1. 41.15 2. 37.96 Range 18–65	*N =* 105 (68.6%) Number in each condition: 1. *N* = 53 (66%) 2. *N* = 52 (71.2%)	Total data sets analysed *N* = 105–89 completed treatment and 16 ITT data sets	Pre and post intervention No follow up
Dixon-Gordon et al. (2015) USA	Pilot study Randomisation: Yes Control group: Yes	1. DBT-ER (ER skills only) 2. DBT-IE (interpersonal effectiveness skills only) 3. Interpersonal and Psycho-Education group (IPE), no DBT skills	6 weeks, no information on session length	Women with Borderline Personality Disorder (BPD) Mean age 34.47 across groups Range 20–60 Ethnicity: white 63.2%, East Asian 21.1%	*N* =19 (100%) Number in each condition: 1. *N* = 7 2. *N* = 6 3. *N* = 6	Total data sets analysed *N* = 19–17 completed treatment and 2 ITT data sets	Pre, mid and post intervention Two month follow-up
Ford et al. (2014) USA	RCT Randomisation: Yes Control group: Yes	1. Trauma Affect Regulation: Guide for Education and Therapy (TARGET) 2. Supportive group therapy	12 weeks, 75 min each session	Women in prison with full or partial Post-traumatic stress disorder (PTSD) Mean age 1. 34.6 2. 38 Range 23–57 Ethnicity: 40% people of colour, 60% white	*N* = 80 (100%) Number in each condition: 1. *N* = 41 2. *N* = 39	Total data sets analysed *N* = 72	Pre and post intervention No follow-up
Gratz & Gunderson (2006) USA	RCT Randomisation: Yes Control group: Yes	1. Acceptance-based emotion regulation group intervention (ERGT) and treatment as usual (TAU) 2. TAU—mixture of individual therapy, self-help, group therapy, other appointments	14 weeks, 90 min each session	Women with BPD who self-harm Mean age 1. 33 2. 33.7 Range 19–58 Ethnicity: 100% white	*N* = 24 (100%) Number in each condition: 1. *N* = 12 2. *N* = 10	Total data sets analysed *N* = 22	Pre and post intervention No follow-up
Gratz & Tull (2011) USA	Before and after Randomisation: No Control group: No	1. ERGT and TAU	14 weeks, 90 min each session	Women who self-harm with either threshold or subthreshold diagnoses of BPD Mean age 1. 34.3 Range 18–50 Ethnicity 87% white, 13% non-white	*N* = 23 (100%) Number in each condition: 1. *N* = 23	Total data sets analysed *N* = 23–19 completed intervention and 4 ITT data sets	Pre and post intervention No follow-up
Gratz et al. (2014) USA	RCT and uncontrolled 9-month follow-up Randomisation: Yes Control group: Yes	1. ERGT and TAU 2. TAU	14 weeks, 90 min each session	Female out-patients who self-harm with either threshold or subthreshold diagnoses of BPD Mean age 1. 33.3 2. 33 Range 18–60 Ethnicity: 1. 16.1% racial/ethnic minority 2. 13.7% racial/ethnic minority	*N* = 61 (100%) Number in each condition: 1. *N* = 31 2. *N* = 30	Total data sets analysed *N* = 61–53 completed intervention and 8 ITT data sets	Pre and post intervention Three and six months follow-up
Holmqvist Larsson et al. (2020) Sweden	Before and after Randomisation: No Control group: No	1. Emotion regulation skills training. Based on ERGT, UP, DBT and ACT	5 weeks, 120 min each session	Female out-patients with eating disorders Mean age 1. 21.41 Range 18–24 Ethnicity: primarily Caucasian	*N* = 39 (100%) Number in each condition: 1. *N* = 39	Total data sets analysed *N* = 29	Pre and post intervention No follow-up
Morvaridi et al. (2019) Iran	Quasi-experimental Randomisation: Yes Control group: Yes	1. Emotional schema therapy (Leahy, 2015) 2. Waitlist	10 weeks, 120 min each session	Women with social anxiety Mean age 1. 23.83 2. 24 Range 18 to 35	*N* = 26 (100%) Number in each condition: 1. *N* = 13 2. *N* = 13	Total data sets analysed *N* = 24	Pre and post intervention No follow-up
Rizvi & Steffel (2014) USA	Before and after Randomisation: No Control group: Yes	1. ER skills training from DBT modules 2. ER and mindfulness skills training from DBT modules	8 weeks, 120 min each session	Undergraduates with significant problems with emotional regulation Mean age no mean provided Range 18–29 Ethnicity: 71% Caucasian, 17% Asian and 13% African American	*N* = 24 (88%) Number in each condition: 1. *N* = 8 2. *N* = 16	Total data sets analysed *N* = 24–17 completed intervention and 7 ITT data sets	Pre, mid and post intervention Three-month follow-up (apart from first group)
Ryan et al. (2021) Ireland	Before and after Randomisation: No Control group: No	1. Living through psychosis (LTP)	8 sessions over 4 weeks. First session was 300 min and subsequent sessions were 180 min	People with a psychotic disorder diagnosis at an independent psychiatric hospital	*N =* 62 (40%) Number in each condition: 1. *N* = 62	Total data sets analysed *N* = 55	Baseline, pre, post and follow-up Four week follow-up
Sahlin et al. (2017) Sweden	Before and after Randomisation: No Control group: No	1. ERGT	14 weeks, 120 min each session	Women at 14 psychiatric outpatient clinics meeting 3 or more diagnostic criteria for BPD and 3 or more episodes of DSH in last 6 months td align="center" valign="middle" style="border-bottom:solid thin">*N* = 95 (100%) Number in each condition: 1. *N* = 95	Total data sets analysed *N* = 95, 74 completed intervention and 21 ITT data sets	Pre and post intervention Six month follow-up	
Varkovitzky et al. (2018) USA	Observational case series Randomisation: No Control: No	1. Unified protocol (UP) for the transdiagnostic treatment of emotional disorders group intervention	16 weeks, 90 min each session	Patients at PTSD outpatient clinic for veterans Mean age 1. 46.65 No range reported Ethnicity: White (65.4%), black (9.6%), Native Hawaiian/Pacific Islander (3.8%), Asian American (3.8%), Latino (19.2%).	*N* = 170 (17%) Number in each condition: 1. *N* = 170	Total data sets analysed *N* = 52	Pre and post intervention No follow-up
Zargar et al. (2019) Iran	RCT Randomisation: Yes Control: Yes	1. ERGT +TAU 2. TAU—methadone (medication) therapy	8 weeks, 120 min each session	Male patients admitted to addiction centre with substance use disorder Mean age 1. 25.70 2. 24.85 Range 20–50	*N* = 34 (0%) Number in each condition: 1. *N* = 17 2. *N* = 17	Total data sets analysed *N* = 30	Pre and post intervention No follow-up

**Table 2:** Summary of treatment effects on ER outcome measure.

Study	ER Measure	Pre Mean (SD)	Post Mean (SD)	Effect Size(s) and/or Summary of Results	Were Changes in ER Accompanied by Other Clinically Relevant Changes?
Studies with active control conditions (*N* = 4)					
Berking et al. (2019) Quality rating: 32/38 (Good)	ERSQ Higher scores indicate better emotional regulation, maximum score of 4	ART 1.72 (0.66) CFC 1.73 (0.63) WLC 1.63 (0.62)	ART 2.18 (0.65) CFC 1.91 (0.77) WLC 1.71 (0.66)	Within Participants *d* = 0.70 ^a^ *p* < 0.0001 *d* = 0.26 ^a^ *p* < 0.001 *d* = 0.13 ^a^ *ns* Between Participants Self-report ERSQ: Significantly greater increase for ART than in WLC: *d* = 0.73, *p* < 0.001. Observer-based ERSQ—ART is associated with a greater increase of emotion regulation skills than WLC: *d* = 0.69, *p* < 0.01. Difference between WLC and CFC: *d* = 0.46, *p* < 0.10 No significant difference between CFC and ART on ERSQ Interaction effects Time x group χ^2^ = 4.27 *p* < 0.001 ART vs. WLC t = 3.75 *p* < 0.001 ART vs. CFC t = 1.39 *ns* CFC vs. WLC t = 2.29 *ns*	Yes—depressive symptoms also reduced—significantly larger reduction in ART compared to WLC. However no significant differences in symptom reduction between ART and CFC.
Dixon-Gordon et al. (2015) Quality rating: 19/38 (Fair)	DERS Lower scores indicate better emotion regulation	DBT-ER 123.86 (28.72) DBT-IE 109.50 (15.86) IPE 108.50 (14.69)	DBT-ER 106.00 (31.13) 2. DBT-IE 106.40 (22.01) IPE 97.00 (17.28)	Within Participants *d* = 0.48 *p* < 0.05 *d* = 0.18 *ns* *d* = 0.75 *ns* Interaction effects Condition x Time interaction was nonsignificant. *d* = 0.85 *ns*	Yes—improvements in a range of other domains: interpersonal skills, distress tolerance, mindfulness, BPD symptoms, depressive symptoms and non suicidal self-injury
Ford et al. (2014) Quality rating: 30/38 (Good)	NMR Higher scores indicate better emotion regulation	TARGET 98.7 (16.2) SGT 104.7 (15.8)	TARGET 105.1 (18.0) SGT 104.7 (15.9)	Within Participants *d* = 0.37 *ns* *d* = 0.00 *ns* Interaction effects Group×Time interaction was not significant: *d* = 0.32 *ns*	Yes—reduction in PTSD and trauma symptoms and general distress (as measured by CORE-OM)
Rizvi & Steffel (2014) Quality rating: 13/38 (Poor)	DERS Lower scores indicate better emotion regulation	DBT-ER 124.88 (14.27) DBT-MF+ER 120.31 (23.16)	DBT-ER 98.54 (18.55) DBT-MF+ER 83.16 (26.54)	Within Participants *d* = 1.59 ^a^ *p* < 0.05 *d* = 1.49 ^a^ *p* < 0.05 Between Participants No significant differences between groups on DERS Interaction effects Time x group interaction: F = 23.80 *p* < 0.001 * *d* = 1.52 * *p* < 0.01 set as significance level due to multiple comparisons	Yes—improvements in depression and stress
Studies with waitlist or TAU control *N* = 4					
Corpas et al. (2021) Quality rating: 29/38 (Good)	Emotion Regulation Questionnaire (ERQ) Measures the use of two ER strategies: Cognitive Reappraisal (R) and Expressive Suppression (S). Higher scores on R indicates better ER, higher scores on S indicate lower ER	UP R 17.62 (5.20) S 11.52 (4.28) TAU R 16.45 (5.20) S 11.40 (3.70)	UP R 23.48 (6.72) S 7.50 (2.30) TAU R 15.12 (5.20) S 12.90 (4.42)	Within Participants *d* = −1.11 *p* < 0.00 *d* = 0.92 *p* < 0.00 *d* = 0.25 *p* < 0.05 *d* = −2.77 *p* < 0.01 Interaction effects For R: t = 7.68 *p* < 0.001 *d* = −1.36 For S: t = 7.68 *p* < 0.001 *d* = 1.32 UP superior to TAU	Yes—reductions on scores of anxiety and reduced number of people meeting criteria for diagnosis for a range of mental health problems.
Gratz et al. (2014) Quality rating: 25/38 (Fair)	DERS Lower scores indicate better emotion regulation	ERGT + TAU 106.81 (21.87) TAU 112.26 (25.31)	ERGT + TAU 95.27 (15.60) TAU 113.62 (15.60)	Within Participants *d* = 0.61 ^a^ *p* < 0.05 *d* = −0.06 ^a^ *ns* Between Participants ERGT superior to TAU *D* = 0.55 *p* < 0.05 ERGT + TAU—29% reliable improvements, 61.3% normal functioning TAU 10% reliable improvement, 23.3% normal functioning Interaction effects Not reported	Yes—significant improvements on deliberate self-harm, BPD symptoms, depression, stress and quality of life
Gratz & Gunderson (2006) Quality rating: 23/38 (Fair)	DERS Lower scores indicate better emotion regulation	ERGT + TAU 127.92 (19.99) TAU 119.90 (20.86)	ERGT + TAU 79.75 (23.97) TAU 115.80 (16.74)	Within Participants *n_p_*^2^ = 0.80 *p* < 0.01. *n_p_*^2^ = 0.07 *ns* Between Participants Significant between group differences *n_p_*^2^ = 0.54, *p* < 0.01 in favour of ERGT The treatment group reached normal levels of functioning on measures of emotion dysregulation (mean DERS among female college students = 77.99) 83% of participants in the treatment group reported reliable improvements in ER Interaction effects Not reported	Yes—positive effects on self-harm, experiential avoidance, BPD symptoms, depression, anxiety and stress
Morvaridi et al. (2019) Quality rating: 11/38 (Poor)	(ERQ) Higher scores on R indicates better ER, higher scores on S indicate lower ER	EST R 20.08 (5.33) S 16 (3.83) WLC R 18.58 (3.60) S 17.58 (3.20)	EST R 33.16 (4.08) S 9.25 (2.70) WLC R 17.75 (3.67) S 18.66 (3.42)	Within Participants *d* = 2.76 ^a^ *p* < 0.05 *d* = 2.04 ^a^ *p* < 0.05 *d* = 0.23 ^a^ *ns* *d* = 0.33 ^a^ *ns* Between Participants There was a significant difference between the EST and WLC in the post-test scores of both ER components, suppression and reappraisal (*p* < 0.001) (*n_p_*^2^ = 0.76 and *n_p_*^2^ = 0.81), with the experimental group showing significantly improved ER. Interaction effects Not reported	Yes—reduced anxiety symptoms
Zargar et al. (2019) Quality rating: 24/38 (Fair)	DERS Lower scores indicate better emotion regulation	ERGT(G) + TAU 104.12 (9.21) TAU 97.65 (5.62)	ERGT(G) + TAU 96.69 (5.38) TAU 73.70 (5.05)	Within Participants *d* = 0.99 ^a^ *p* < 0.05 *d* = 4.48 ^a^ *p* < 0.05 Between Participants Significant difference between ERGT(G) and TAU and TAU, ERGT(G) superior. *n*^2^ = 0.81, *p* = 0.001 Interaction Effects Not reported	Yes—improved martial adjustment, decreased cravings
Studies with no control *N* = 5					
Bacon et al. (2018) Quality rating: 17/38 (Poor)	DERS Lower scores indicate better emotion regulation	ERG 132.19 (19.73)	ERG 108.38 (25.56)	Within Participants *d* = 1.04, *p* < 0.001 51% demonstrated reliable change and 43% demonstrated clinical significant change	Yes—significant improvements in self-efficacy and mental wellbeing
Gratz & Tull (2011) Quality rating: 18/38 (Poor)	DERS Lower scores indicate better emotion regulation	ERGT 110.74 (22.13)	ERGT 80.32 (23.31)	Within Participants *n_p_*^2^ = 0.67, *p* = < 0.05 63.2% reliable change 84.2% normal functioning	Yes—improvements in deliberate self-harm, experiential avoidance and psychiatric symptoms
Holmqvist Larsson et al. (2020) Quality rating: 21/38 (Fair)	DERS Lower scores indicate better emotion regulation	ERST 112.19 (16.38)	ERST 93.56 (16.42)	Within Participants *d* = 1.14 *p* < 0.001	Yes—improvement in alexithymia and reduction in eating disorder symptoms and clinical impairment
Ryan et al. (2021) Quality rating: 17/38 (Poor)	DERS Lower scores indicate better emotion regulation	LTP 98.4 (21.4)	LTP 92.3 (21.8)	Within Participants *d* = 0.28 ^a^ *p* < 0.00	Yes—reduction in hallucinations, delusion severity and distress, increase in recovery scale and mindfulness scale
Sahlin et al. (2017) Quality rating: 23/38 (Fair)	DERS Lower scores indicate better emotion regulation	ERGT 125.98 (19.37)	ERGT 104.66 (27.40)	Within Participants *d* = 0.91 *p* < 0.001	Yes—reduction in self-harm frequency, other self-destructive behaviours and general psychiatric symptoms
Varkovitzky et al. (2018) Quality rating: 13/38 (Poor)	DERS Lower scores indicate better emotion regulation	UP 125.39 (19.98)	UP 109.12 (29.35)	Within Participants Hedges’s *g* 0.64, *p* < 0.001	Yes—improvements in PTSD and depressive symptoms

Notes: ^a^ = Effect size calculated by first author; ART = Affect regulation training; CFC = Common factors control condition; SGT = Supportive group therapy; TAU = Treatment as normal; WLC = Waitlist control condition; ERGT = Emotion regulation group therapy; ERGT(G) = Emotion regulation group therapy based on Gross model; ERG = Emotional resources group; UP = Unified protocol for transdiagnostic treatment of emotional disorders; ERST = Emotion regulation skills training; EST = Emotional schema therapy; DBT-ER = Dialectical Behaviour Therapy–Emotion regulation skills module; DBT-MF+ER = Dialectical Behaviour Therapy–Mindfulness and emotion regulation skills modules; DBT-IE = Dialectical Behaviour Therapy–Interpersonal effectiveness skills module; IPE = psychoeducation group; LTP = Living Through Psychosis group programme; DERS = Difficulties in emotion regulation scale; ERSQ = Emotion Regulation Skills Questionnaire; NMR = Generalized Expectancies for Negative Mood Regulation; *ns* = not significant, *p* > 0.05. The effect sizes for Cohen’s d and Hedges g are the following: 0.2 represents a “small” effect size, 0.5 represents a “medium” effect size, and 0.8 represents a “large” effect size. The effect sizes for partial eta squared *np*^2^ and eta squared *n*^2^ are the following: 0.01 represents a small effect size, 0.06 represents a moderate effect size, and 0.14 represents a large effect size.

## Data Availability

Not applicable.

## Appendix A

**Table A1:** Downs and Black Modified Checklist.

Reporting	
1. Is the hypothesis/aim/objective of the study clearly described?	Yes = 1, No = 0
2. Are the main outcomes clearly described in the Introduction or Methods? No if main outcome measures first mentioned in results.	Yes = 1, No = 0
3. Are characteristics of the patients included in the study clearly described? Yes if inclusion and exclusion criteria clearly described.	Yes = 1, No = 0
4. Are the interventions clearly described? Yes if it clear what intervention is being delivered and an attempt has been made to describe the content of the intervention and what ER skills are used.	Yes = 1, No = 0
5. Are there clear theory to practise links that justify the use of the specific intervention to improve ER? ^a^ Yes if there is a described ER theory and explicit links to the intervention used. Partially if an ER theory is presented but no or little attempt is made to link it to the intervention used.	Yes = 2, Partially = 1, Unclear = 0, No = 0
6. Are any other interventions participants are receiving clearly described? ^a^ e.g., other therapeutic input	Yes = 1, Unclear = 0, No = 0
7. Are the distributions of principal confounders in each group of subjects clearly described? If the answer to 6. is unclear or no, the answer to this cannot be Yes.	Yes = 2, Partially = 1, No = 0
8. Are the main findings of the study clearly described? Yes if at least pre and post measures of ER are reported.	Yes = 1, No = 0
9. Does the study estimate random variability in data for main outcomes? E.g., standard error, standard deviation, interquartile range or confidence interval reported.	Yes = 1, No = 0
10. Have all the important adverse events consequential to the intervention been reported? This should be answered yes if the study demonstrates that there was a comprehensive attempt to measure adverse events. (A list of possible adverse events is provided).	Yes = 1, No = 0
11. Have characteristics of patients lost to follow-up been described? This should be answered yes where there were no losses to follow-up or where losses to follow-up were so small that findings would be unaffected by their inclusion. This should be answered no where a study does not report the number of patients lost to follow-up.	Yes = 1, No = 0
12. Have actual probability values and effect sizes been reported for the main outcomes except probability <0.001?	Yes = 1, No = 0
13. Is the source of funding clearly stated? ^a^	Yes = 1, No = 0
**External validity**	
14. Were subjects who were asked to participate in the study representative of the entire population recruited? The study must identify the source population for patients and describe how the patients were selected. Patients would be representative if they comprised the entire source population, an unselected sample of consecutive patients, or a random sample. Random sampling is only feasible where a list of all members of the relevant population exists. Where a study does not report the proportion of the source population from which the patients are derived, the question should be answered as unable to determine	Yes = 1, No = 0, Unable to determine = 0
15. Were those subjects who were prepared to participate representative of the recruited population? The proportion of those asked who agreed should be stated. Validation that the sample was representative would include demonstrating that the distribution of the main confounding factors was the same in the study sample and the source population.	Yes = 1, No = 0, Unable to determine = 0
16. Were the staff, places, and facilities where the patients were treated, representative of the treatment the majority of patients receive? For the question to be answered yes the study should demonstrate that the intervention was representative of that in use in the source population. The question should be answered no if, for example, the intervention was undertaken in a specialist centre unrepresentative of the hospitals most of the source population would attend.	Yes = 1, No = 0, Unable to determine = 0
**Internal Validity—Bias**	
17. Was an attempt made to blind those measuring the main outcomes? For studies where the patients would have no way of knowing which intervention they received, this should be answered yes.	Yes = 1, No = 0, Unable to determine = 0
18. Were analyses planned apriori, pre-registered on a public website, and the analysis plan followed? ^a^ Any analyses that had not been planned at the outset of the study should be clearly indicated. If no retrospective unplanned subgroup analyses were reported, then answer yes	Yes = 1, No = 0, Unable to determine = 0
19. Was the time period between intervention and outcome the same for intervention and control groups or adjusted for? Where follow-up was the same for all study patients the answer should yes. If different lengths of follow-up were adjusted for by, for example, survival analysis the answer should be yes. Studies where differences in follow-up are ignored should be answered no.	Yes = 1, No = 0, Unable to determine = 0
20. Were the statistical tests used to assess main outcomes appropriate? The statistical techniques used must be appropriate to the data. For example nonparametric methods should be used for small sample sizes. Where little statistical analysis has been undertaken but where there is no evidence of bias, the question should be answered yes.	Yes = 1, No = 0, Unable to determine = 0
21. Was compliance with the interventions reliable? ^a^ E.g., did participants attend all sessions, and if not, was this reported and taken into account. Where there was non compliance with the allocated treatment or where there was contamination of one group, the question should be answered no.	Yes = 1, No = 0, Unable to determine = 0
22. Is there evidence that the intervention was delivered as intended? ^a^ E.g., was there a measure of facilitator fidelity, were facilitators adequately trained and supervised	Yes = 1, No = 0, Unable to determine = 0
23. Were main outcome measures used accurate? (valid and reliable) For studies where the outcome measures are clearly described, the question should be answered yes. For studies which refer to other work or that demonstrates the outcome measures are accurate, the question should be answered as yes	Yes = 1, No = 0, Unable to determine = 0
**Internal Validity—Confounding (Selection Bias)**	
24. Were patients in different intervention groups recruited from the same population? For example, patients for all comparison groups should be selected from the same hospital. The question should be answered unable to determine for cohort and case control studies where there is no information concerning the source of patients included in the study	Yes = 1, No = 0, Unclear = 0
25. Were study subjects in different intervention groups recruited over the same period of time? For a study which does not specify the time period over which patients were recruited, the question should be answered as unable to determine	Yes = 1, No = 0, Unclear = 0
26. Were study subjects randomized to intervention groups? Studies which state that subjects were randomised should be answered yes except where method of randomisation would not ensure random allocation. For example alternate allocation would score no because it is predictable	Yes = 1, No = 0, Unclear = 0
27. Was an appropriate method of randomisation described? ^a^ Yes if a method was described unless it would not lead to truly random allocation—e.g., alternate, alphabetical, date of birth	Yes = 1, No = 0, Unclear = 0
28. Were attempts made to assess groups equivalence at baseline? ^a^ If groups are not equivalent, are there statistical techniques introduced to control for between group differences at baseline? E.g., baseline scores on measures, age, gender, diagnosis, number in each group	Yes = 1, No = 0, Unclear = 0
29. Are any other interventions participants are receiving adequately controlled for? E.g., are they equivalent across groups and if not techniques introduced to control for this	Yes = 1, Unclear = 0, No = 0
30. Was there an adequate control group? ^b^ No if no control or waitlist/TAU control. Yes if attempts are made to control for common therapeutic/group factors or comparative group intervention used	Yes = 1, No = 0, Unclear = 0
31. Was the randomized intervention assignment concealed from patients and staff until recruitment was complete? All non-randomised studies should be answered no. If assignment was concealed from patients but not from staff, it should be answered no.	Yes = 1, No = 0, Unclear = 0
32. Was there adequate adjustment for confounding in the analyses from which main findings were drawn? This question should be answered no for trials if: the main conclusions of the study were based on analyses of treatment rather than intention to treat; the distribution of known confounders in the different treatment groups was not described; or the distribution of known confounders differed between the treatment groups but was not taken into account in the analyses. In nonrandomised studies if the effect of the main confounders was not investigated or confounding was demonstrated but no adjustment was made in the final analyses the question should be answered as no	Yes = 1, No = 0, Unclear = 0
33. Were losses of patients to follow-up taken into account? If the numbers of patients lost to follow-up are not reported, the question should be answered as unable to determine. If the proportion lost to follow-up was too small to affect the main findings, the question should be answered yes.	Yes = 1, No = 0, Unclear = 0
34. Are attempts made to use intention-to-treat analysis or similarly robust methods to manage losses to follow up? ^a^ Yes if ITT is used or the use of a similar method of handling missing data is described and defended. Partially if mentioned but no justification of choice of handling missing data.	Yes = 2, Partially = 1, Unclear = 0, No = 0
**Power ^a^**	
35. Was the study sufficiently powered to detect clinically important effects where probability value for a difference due to chance is <5%? Yes if power is discussed and clearly taken into account apriori	Yes = 1, No = 0, Unclear = 0

^a^ denotes additional or modified questions for the purposes of this systematic review. ^b^ additional question added from MINORS [98]. Total possible for RCT: 38. Total possible for non-randomised with comparator/control group: 35. Total possible for non-randomised with no comparator/control group: 28. Score ranges are given corresponding quality levels: excellent (35–38); good (26–34); fair (21–25); and poor (≤19) modified from [60] quality level ratings.
